# Benign Enchondroma in a 40-Year-Old Female: Emphasizing the Importance of Early 18F-FES PET/CT Utilization

**DOI:** 10.1155/crra/9992200

**Published:** 2025-08-04

**Authors:** Tamer M. Dawud, Abdullah T. Dawud

**Affiliations:** ^1^Department of Nuclear Medicine, Cleveland Clinic Imaging Institute, Cleveland, Ohio, USA; ^2^Independent Researcher, Ellicott City, Maryland, USA

**Keywords:** 18F-FES PET/CT, biopsy, bone metastasis, breast cancer, enchondroma, Tc-99m MDP

## Abstract

A 40-year-old female with estrogen receptor–positive breast cancer underwent an initial staging using a technetium-99m methylene diphosphonate (Tc-99m MDP) bone scan, which revealed abnormal uptake in the femur without a patient history of prior trauma or associated symptoms. Subsequently, an MRI confirmed the presence of a well-defined lesion in the upper left femur. To rule out metastatic disease, an 18F-fluoroestradiol (FES) PET/CT was performed, demonstrating no ER expression. Following the PET/CT, a biopsy confirmed the presence of an enchondroma. This case underscores the importance of early utilization of 18F-FES PET/CT in breast cancer staging to minimize unnecessary additional procedures/imaging.

**Summary**

• Question: Should 18F-FES PET/CT replace bone scans as the initial imaging modality for evaluating suspected metastases in ER-positive breast cancer patients?
• Pertinent findings: This diagnostic case study of a 40-year-old patient with ER-positive breast cancer demonstrated that negative 18F-FES PET/CT uptake (despite positive Tc-99m MDP bone scan) correctly predicted a benign enchondroma, as confirmed by MRI and biopsy.
• Implications for patient care: Early integration of 18F-FES PET/CT in the diagnostic workflow may reduce unnecessary procedures and improve staging efficiency for ER-positive breast cancer patients with indeterminate skeletal findings.

## 1. Introduction

Breast cancer is the most commonly diagnosed malignancy in women in the United States [1], with bone being a frequent metastatic site [2]. Accurate distinction between benign and malignant bone lesions is critical for appropriate staging and treatment planning. Treatment often involves advanced imaging modalities that detect potential metastases and guide treatment decisions. Among these, technetium-99m methylene diphosphonate (Tc-99m MDP) bone scans are widely used to detect skeletal metastases due to their high sensitivity [3]. However, their limited specificity complicates the differentiation between malignant metastases and benign conditions. As noted by Cook, “Specificity for detecting metastatic lesions may also be limited without further morphological imaging as several benign processes can cause focal uptake in the skeleton” [4]. This diagnostic uncertainty often necessitates additional imaging—such as magnetic resonance imaging (MRI) and positron emission tomography/computed tomography (PET/CT)—or biopsy to confirm the nature of suspicious lesions.

18F-fluoroestradiol (FES) PET/CT has emerged as a valuable imaging modality for evaluating estrogen receptor (ER)–positive breast cancer, as it specifically targets ER expression in tumor cells [5]. Meta-analyses report 18F-FES PET/CT sensitivity of 71%–82% [6, 7], while a more recent 2022 study of 200 patients demonstrated improved sensitivity of 95% [8]. The radiotracer's specificity was recently reported at 98% in a recent meta-analysis [9]. However, 18F-FES PET/CT cannot reliably identify benign bone lesions such as enchondromas [6].

Enchondromas are benign cartilaginous tumors that typically arise in the medullary cavity of long bones, most frequently in the hands and feet [10]. Typically asymptomatic, they are often incidental findings on imaging performed for unrelated reasons. Their clinical and radiological features may mimic those of malignant bone lesions, complicating the diagnostic process [11].

## 2. Case Report

**Figure 1:**
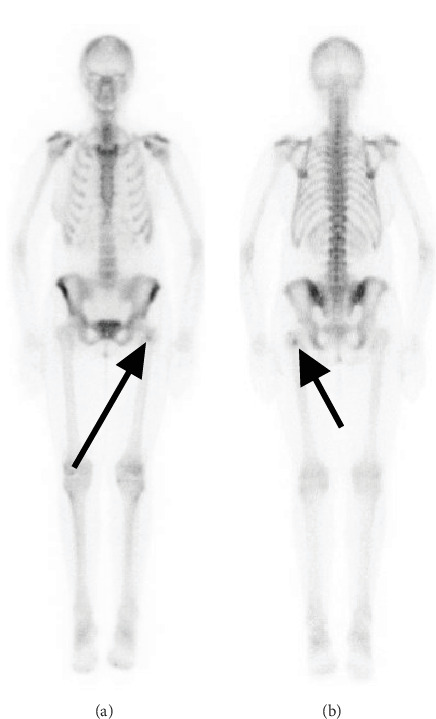
(a) Anterior (arrow) and (b) posterior (arrow) whole-body bone scan with intense radiotracer uptake in the upper left femur.

**Figure 2:**
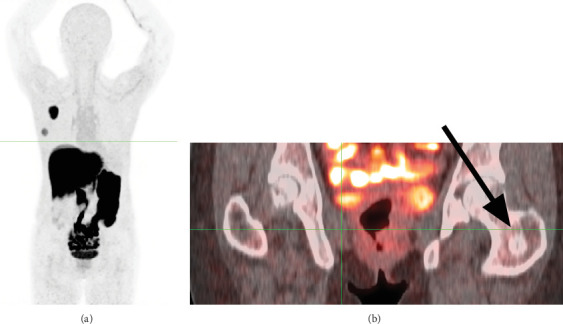
(a) Maximum intensity projection (MIP) 18F-FES PET and (b) fused 18F-FES PET/CT images (arrow) exhibiting no radiotracer uptake in the upper left femur.

**Figure 3:**
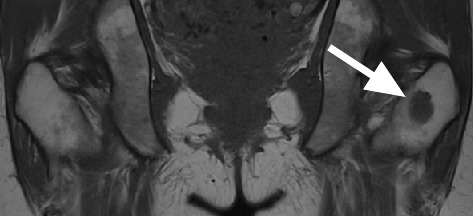
Coronal T1-weighted MRI image demonstrating a well-defined lesion (white arrow) in the upper left femur.

A 40-year-old female with a recent diagnosis of ER-positive breast cancer underwent an initial staging, including a Tc-99m MDP bone scan. The bone scan revealed an area of abnormal radiotracer uptake in the proximal left femur (Figure 1). The patient reported no history of trauma, pain, or other symptoms related to the femur. Subsequently, imaging with 18F-FES PET/CT revealed no radiotracer uptake in the femoral lesion (Figure 2), consistent with the absence of ER expression. Concurrently, MRI confirmed the presence of this low-signal-intensity T1 lesion (1.8 × 1.6 × 2.3 cm shown in Figure 3). Given the discordance between the abnormal bone scan and the absence of 18F-FES PET/CT uptake, a biopsy was performed to rule out metastasis definitively. The examination of the femoral lesion confirmed the diagnosis of an enchondroma. The patient's breast cancer was staged as T2N0M0 due to the absence of metastasis. No further intervention was required for the enchondroma, and she was referred to oncology for further management, including surgical resection of the primary tumor.

## 3. Discussion

18F-FES is approved by the US Food and Drug Administration as a diagnostic agent for “the detection of ER-positive lesions as an adjunct to biopsy in patients with recurrent or metastatic breast cancer” [12]. Following these guidelines, this case utilized 18F-FES PET/CT after a bone scan initially raised suspicion for metastasis. Although the FES PET/CT ultimately showed no uptake in the lesion, an MRI and biopsy were subsequently performed to confirm the lesion's benign nature. Had the FES PET/CT been incorporated earlier in the diagnostic workup, the absence of ER expression might have provided sufficient evidence against metastatic disease, potentially eliminating the need for additional imaging and an invasive biopsy. This case underscores the value of incorporating FES PET/CT earlier in the diagnostic process, which could reduce unnecessary procedures, optimize resource utilization, and ultimately improve diagnostic efficiency in similar scenarios.

## Data Availability

Data sharing is not applicable to this article as no datasets were generated or analyzed during the current study.
